# Two cases of CRPC with *BRCA* mutation treated by olaparib after favorable response to cisplatin

**DOI:** 10.1002/iju5.12543

**Published:** 2022-10-09

**Authors:** Yoshiyuki Miyazawa, Takanori Shimizu, Yoshitaka Sekine, Seiji Arai, Akira Ohtsu, Yuji Fujizuka, Masashi Nomura, Hidekazu Koike, Hiroshi Matsui, Kazuhiro Suzuki

**Affiliations:** ^1^ Department of Urology Gunma University Graduate School of Medicine Gunma Japan

**Keywords:** prostate cancer, castration‐resistant prostate cancer, neuroendocrine prostate cancer, poly(adenosine diphosphate‐ribose) polymerase, BRCA

## Abstract

**Introduction:**

Several prostate cancers carry homologous recombination repair mutations that respond to olaparib. Because of the mechanism, the efficacy of platinum‐based therapy can be used to predict the efficacy of poly(adenosine diphosphate‐ribose) polymerase inhibitors such as olaparib.

**Case presentation:**

We experienced two neuroendocrine prostate cancer patients who achieved a response duration of more than 1 year with platinum‐based therapy. Case 1 had a *BRCA2* mutation in the germline and case 2 had a *BRCA2* mutation in a somatic chromosome only. Both patients responded well to olaparib.

**Conclusion:**

Cisplatin and olaparib may overlap in response due to their medicinal action. It may be useful to consider genetic testing in some CRPC patients who have responded to cisplatin.

**Abbreviations & Acronyms**
AR: androgen receptor
CAB: combined androgen blockade
CRPC: castration‐resistant prostate cancer
GS: Gleason score
NEPC: neuroendocrine prostate cancer
NSE: neuron‐specific enolase
OS: overall survival
PARP: poly(adenosine diphosphate‐ribose) polymerase
PC: prostate cancer
PSA: prostate‐specific antigen

**Keynote message**

Many castration‐resistant prostate cancer patients who convert to neuroendocrine prostate cancer cases have a poor prognosis. However, genetic testing should be considered in neuroendocrine prostate cancer patients who might benefit from platinum‐based therapy.

## Introduction

Prostate cancer (PC) can be effectively treated it with androgen deprivation therapy, but some cases have been reported to develop castration‐resistant prostate cancer (CRPC). Some of these patients may undergo neuroendocrine differentiation that is not mediated by androgen receptor (AR) signaling, resulting in neuroendocrine prostate cancer (NEPC). NEPC is successfully treated with a platinum‐based regimen similar to small cell lung cancer. We experienced two cases in which a *BRCA* mutation was identified after a successful platinum regimen and the subsequent response to olaparib was confirmed.

## Case presentation

### Case 1

**Fig. 1:**
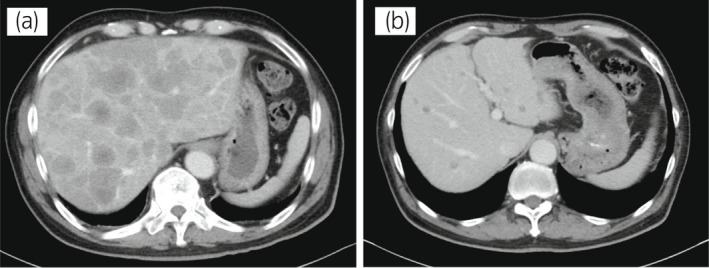
Case 1. Abdominal CT images before and after three courses of cisplatin and etoposide therapy. (a) Before treatment and (b) after the treatment courses.

**Fig. 2:**
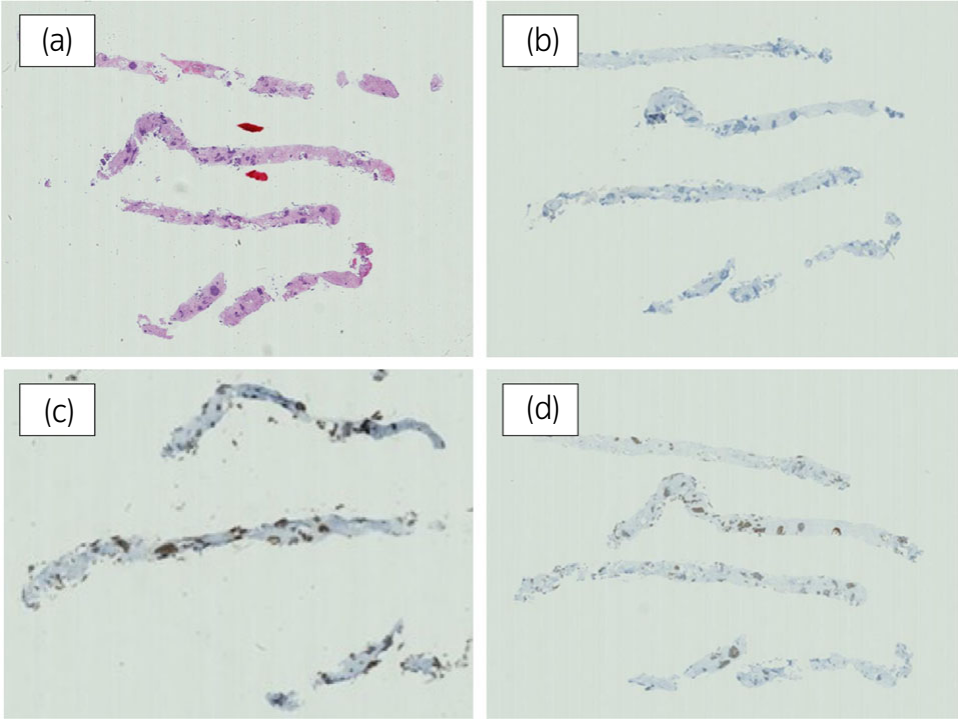
Case 1. The pathological results of the bone metastasis site biopsy. Immunohistochemistry was negative for PSA and positive for chromogranin A and synaptophysin. (a) Hematoxylin–eosin staining, (b) PSA, (c) chromogranin A, (d) synaptophysin.

**Fig. 3:**
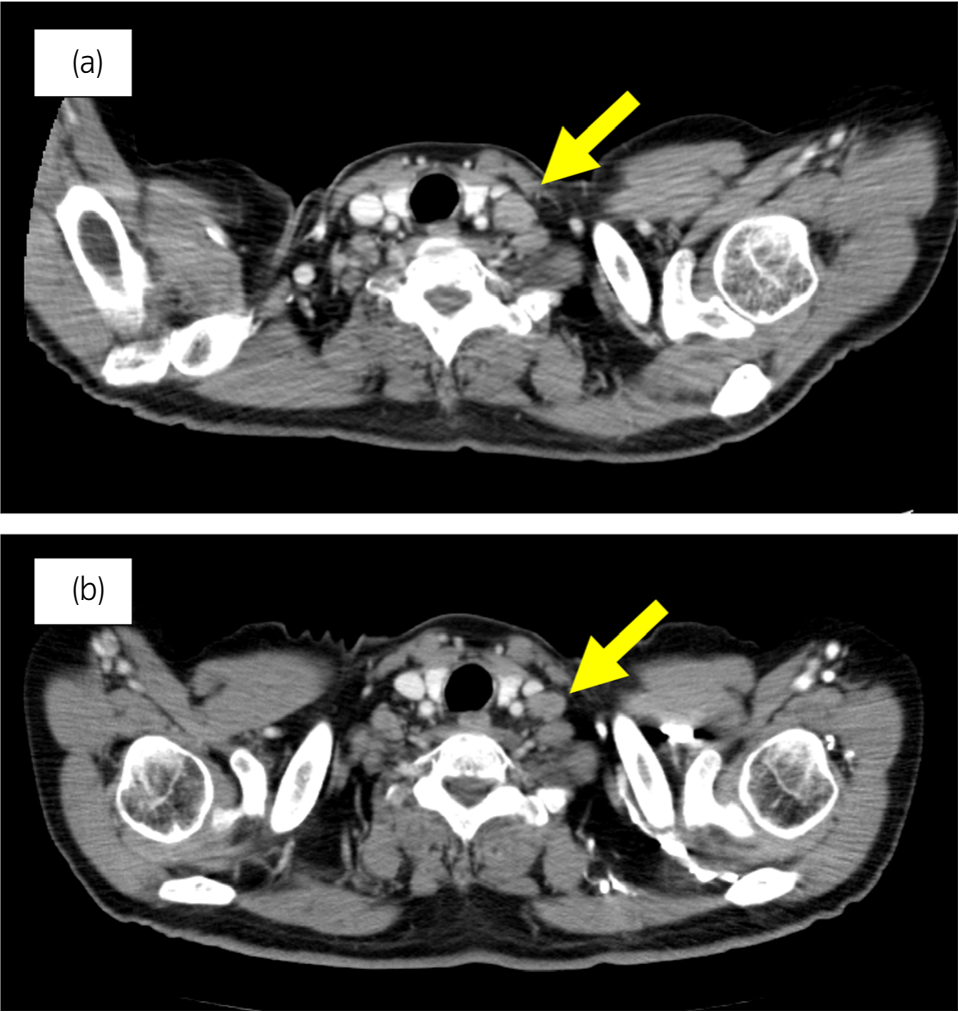
Case 1. CT images of the subclavian lymph node metastases before and 3 months after initiating olaparib. (a) Before treatment and (b) 3 months after olaparib treatment. Yellow arrow shows metastatic lymph node.

The 70‐year‐old man was diagnosed with metastatic PC, cT3bN1M1b, Gleason Score (GS) of 4 + 5 = 9, prostate‐specific antigen (PSA) level of 40.8 ng/ml with metastasis of thoracic vertebra 3 years ago. Combined androgen blockade (CAB) therapy and proton radiation therapy (78.0 Gy/39 fr) to the prostate were performed. The PSA level decreased to <0.01 ng/ml, and the CAB was stopped after 2.5 years. Five months after stopping the CAB, he presented back pain with PSA <0.01 ng/ml. A computed tomography (CT) scan detected multiple liver, bone, and lymph node metastases with a high neuron‐specific enolase (NSE) level of 171 ng/ml (Fig. 1a). A biopsy of the bone metastases revealed the diagnosis of NEPC (Fig. 2), and the patient was referred to our hospital. We treated with cisplatin, etoposide, and leuprorelin. After the third course, the metastatic sites shrunk significantly (Fig. 1b). Cisplatin and etoposide therapy was administered for 1 year, but the patient requested that the medication be stopped due to neuropathy. He was started on enzalutamide. After 3 months of treatment, the patient developed back pain and an enlarged left subclavian lymph node (Fig. 3a). Liver metastases remained unchanged, shrinking after cisplatin treatment. Genetic testing by blood test (BRCAnalysis®︎) confirmed *BRCA2* mutation, so we administered olaparib. Three months after, the back pain improved, and the lymph node metastasis had shrunk and was determined to be partial response (PR) (Fig. 3b). Liver metastatic lesions remained unchanged.

### Case 2

**Fig. 4:**
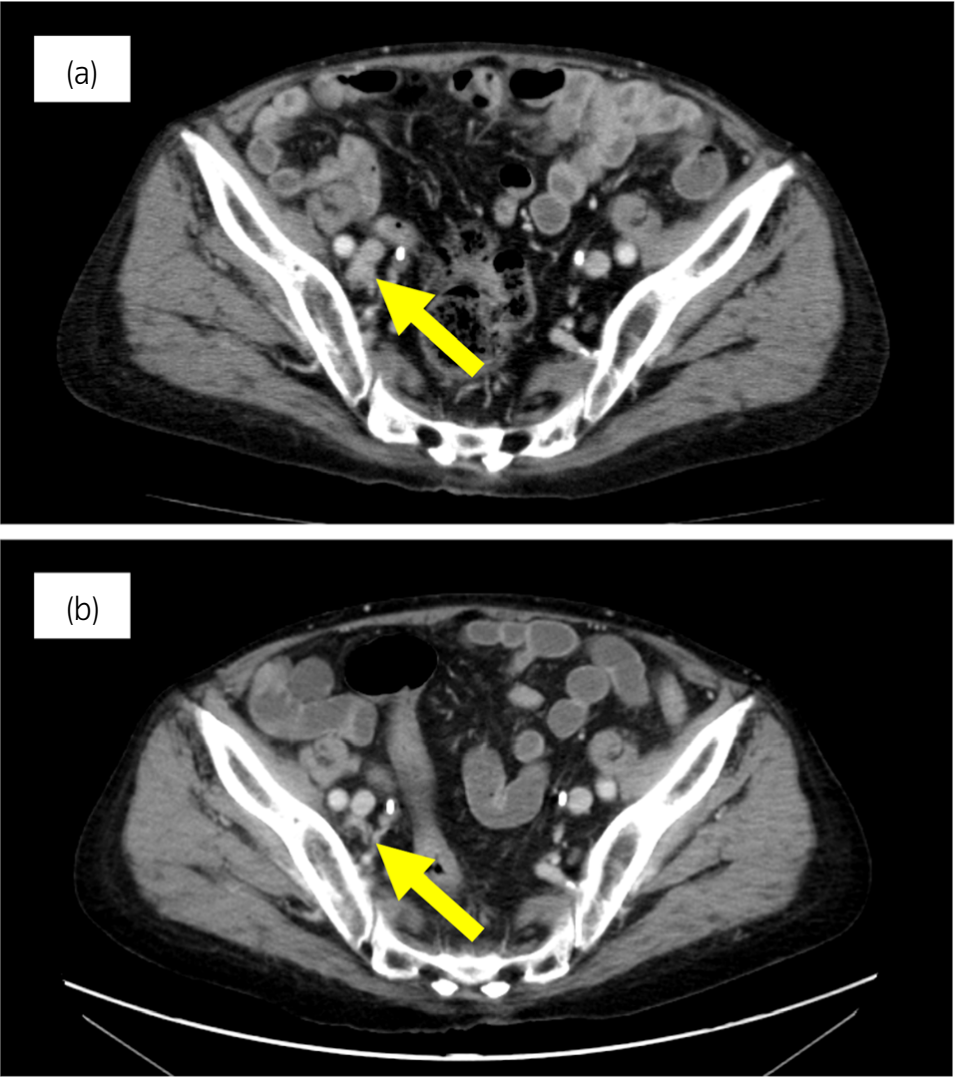
Case 2: CT images of the pelvic lymph nodes before olaparib treatment and 3 months after treatment. (a) Before treatment and (b) 3 months after olaparib treatment. Yellow arrow shows metastatic lymph node.

A 78‐year‐old man was diagnosed with cT3bN0M0, GS of 4 + 4 = 8, PSA level of 15.2 ng/ml 7 years ago, and underwent prostatectomy. Six months after, PSA increased and androgen deprivation therapy was initiated. Two years later, bicalutamide was started for CRPC. Five months after administering bicalutamide, the patient was referred to our hospital. Enzalutamide, docetaxel, and cabazitaxel were administered in sequence over the next 2 years. MRI prior to docetaxel showed iliac bone metastasis, which disappeared with docetaxel and cabazitaxel treatment. Six months after starting cabazitaxel, he developed bilateral pelvic lymph node enlargement and metastatic tumor was found on the posterior wall of the bladder. Tissue samples were obtained from the metastatic tumor in bladder during transurethral resection (TUR) performed. A pathological examination revealed positive findings for chromogranin A and an elevated NSE level, so we diagnosed NEPC. Carboplatin and etoposide combination therapy shrank the tumor. Thereafter, 17 courses were administered over 24 months, and the disease was controlled. Chemotherapy was stopped due to neuropathy and fatigue. Subsequently, the patient showed progression of pelvic lymph node metastasis (Fig. 4a). Genetic testing by blood test (BRCAnalysis®︎) was negative for *BRCA* mutations. A multi‐gene cancer panel test (FoundationOne®︎CDx) was performed using bladder metastatic tissue collected at the time of TUR. The results showed that the patient was positive for a somatic *BRCA2* mutation. The lymph node shrank from 21.0 mm to 7.5 mm over the course of 3 months after olaparib administration (Fig. 4b). PSA decreased from 1.42 to 0.07 ng/ml and NSE from 22.3 to 11.8 ng/ml.

## Discussion

NEPC is an aggressive variant characterized by low or absent AR expression and gain of the neuroendocrine phenotype, but it is not responsive to therapies targeting AR signaling. De novo NEPC accounts for <2% of all prostate cancers, but treatment‐induced NEPC occurs in 10–17% of patients with CRPC as a result of transdifferentiation.^1^ The incidence of treatment‐related NEPC has been rapidly rising due to the increased use of potent AR pathway inhibitors.^2^ A recent autopsy series revealed that 25% of the patients dying from CRPC with signs of NEPC.^3^

Platinum‐based chemotherapy is usually used to treat NEPC. Several studies have shown platinum‐based chemotherapy results in response rates of 8.9–41%, progression‐free survival of 5.8 months, and overall survival of 9.6–12 months.^4^, ^5^, ^6^ In our two cases, case 1 responded for more than 12 months and case 2 was treated for 24 months. Although it is difficult to directly compare, our NEPC cases in which platinum therapy were effective for relatively long time.

CRPC patients with deleterious alterations in genes involved in homologous recombination repair such as *BRCA1*/*2* have more aggressive disease than those with proficient homologous recombination repair.^7^, ^8^ Men who were initially assigned to receive olaparib had a significantly longer duration of overall survival than those who were assigned to receive enzalutamide or abiraterone among metastatic CRPC with at least one mutation in *BRCA1*, *BRCA2*, or *ATM*.^7^ Olaparib is also approved for ovarian and breast cancer. Moore et al. evaluated the efficacy of olaparib as maintenance therapy in patients with advanced ovarian cancer who had a complete or partial response to platinum‐based therapy and had *BRCA1*, *BRCA2*, or both mutations. The risk of disease progression or death was 70% lower with olaparib than with a placebo.^9^ Based on these results, olaparib is being used as maintenance therapy in platinum responders. Platinum drugs induce a type of DNA damage called DNA interstrand crosslinking, which induces cell death. Repair of this damage requires homologous recombination repair, which does not occur in patients with *BRCA1* and *BRCA2* mutations; thus, platinum drugs are effective. We speculated that BRCA1/2 mutations might be associated with a high frequency in NEPC cases that responded to platinum therapy. However, we considered the following two points to be important. First, according to the previous reports, the frequency of HRR mutation including BRCA 1/2 mutations in treatment‐related (induced) NEPC patients is less than 20%.^10^ Kosaka et al. reported the first report of BRCA2 mutations in Japanese NEPC cases in 2019,^11^ but the details in Japanese cases are not clear. It will be necessary to verify whether BRCA1/2 mutations are more frequent in NEPC patients who respond to platinum drugs. Second, in both of our cases, the patient had to stop the platinum regimen due to side effects during the response, and then olaparib was administered, which may have resulted in a response. Considering the mechanism of action, it is possible that cross‐resistance may exist after a platinum failure. Based on our present two cases, it may be useful to actively consider genetic testing in some patients with NEPC who have undergone relatively effective platinum therapy.

## Author contributions

**Yoshiyuki Miyazawa:** Conceptualization; data curation; formal analysis; investigation; resources; validation; writing – original draft. **Takanori Shimizu:** Validation. **Yositaka Sekine:** Supervision; validation. **Seiji Arai:** Supervision. **Akira Ohtsu:** Validation. **Yuji Fujizuka:** Supervision. **Masashi Nomura:** Supervision. **Hidekazu Koike:** Validation. **Hiroshi Matsui:** Supervision. **Kazuhiro Suzuki:** Supervision; writing – review and editing.

## Conflict of interest

Kazuhiro Suzuki has potential financial conflicts of interest as below, Consultancies: Astellas Pharma, Sanofi, Janssen, Bayer, Takeda Pharmaceutical, Daiichi‐Sankyo, AstraZeneca, Grants received: Astellas Pharma, Ono Pharmaceutical, Takeda Pharmaceutical, Daiichi‐Sankyo. All other authors have no conflict of interest exists.

## Approval of the research protocol by an Institutional Reviewer Board

Not applicable.

## Informed consent

We obtained informed consent from all patients.

## Registry and the registration No. of the study/trial

Not applicable.

## References

[1] Wang Y , Wang Y , Ci X *et al*. Molecular events in neuroendocrine prostate cancer development. Nat. Rev. Urol. 2021; 18: 581–96. 34290447 10.1038/s41585-021-00490-0 PMC10802813

[2] Akamatsu S , Inoue T , Ogawa O , Gleave ME . Clinical and molecular features of treatment‐related neuroendocrine prostate cancer. Int. J. Urol. 2018; 25: 345–51. 29396873 10.1111/iju.13526

[3] Aparicio A , Logothetis CJ , Maity SN . Understanding the lethal variant of prostate cancer: power of examining extremes. Cancer Discov. 2011; 1: 466–8. 22586648 10.1158/2159-8290.CD-11-0259 PMC4133693

[4] Culine S , El Demery M , Lamy PJ *et al*. Docetaxel and cisplatin in patients with metastatic androgen independent prostate cancer and circulating neuroendocrine markers. J. Urol. 2007; 178: 844–8. 17631339 10.1016/j.juro.2007.05.044

[5] Fléchon A , Pouessel D , Ferlay C *et al*. Phase II study of carboplatin and etoposide in patients with anaplastic progressive metastatic castration‐resistant prostate cancer (mCRPC) with or without neuroendocrine differentiation: results of the French Genito‐urinary tumor group (GETUG) P01 trial. Ann. Oncol. 2011; 22: 2476–81. 21436186 10.1093/annonc/mdr004

[6] Papandreou CN , Daliani DD , Thall PF *et al*. Results of a phase II study with doxorubicin, etoposide, and cisplatin in patients with fully characterized small‐cell carcinoma of the prostate. J. Clin. Oncol. 2002; 15: 3072–80. 10.1200/JCO.2002.12.065 12118020

[7] Hussain M , Mateo J , Fizazi K *et al*. Survival with olaparib in metastatic castration‐resistant prostate cancer. N. Engl. J. Med. 2020; 383: 2345–57. 32955174 10.1056/NEJMoa2022485

[8] Annala M , Struss WJ , Warner EW *et al*. Treatment outcomes and tumor loss of heterozygosity in germline DNA repair deficient prostate cancer. Eur. Urol. 2017; 72: 34–42. 28259476 10.1016/j.eururo.2017.02.023

[9] Moore K , Colombo N , Scambia G *et al*. Maintenance olaparib in patients with newly diagnosed advanced ovarian cancer. N. Engl. J. Med. 2018; 379: 2495–505. 30345884 10.1056/NEJMoa1810858

[10] Conteduca V , Oromendia C , Eng KW *et al*. Clinical features of neuroendocrine prostate cancer. Eur. J. Cancer 2019; 121: 7–18. 31525487 10.1016/j.ejca.2019.08.011 PMC6803064

[11] Kosaka T , Hongo H , Aimono E *et al*. A first Japanese case of neuroendocrine prostate cancer accompanied by lung and brain metastasis with somatic and germline BRCA2 mutation. Pathol. Int. 2019; 69: 715–20. 31631483 10.1111/pin.12860 PMC6972566

